# Supplementary material for: Overexpression of the chloroplastic 2-oxoglutarate/malate transporter disturbs carbon and nitrogen homeostasis in rice
Source: J Exp Bot. 2020 Jul 25;72(1):137–52. doi: 10.1093/jxb/eraa343 (PMC7816853; doi:10.1093/jxb/eraa343)
Supplement: eraa343_suppl_Supplementary_Figures_Tables [file eraa343_suppl_supplementary_figures_tables.pdf]

Supplementary Figures

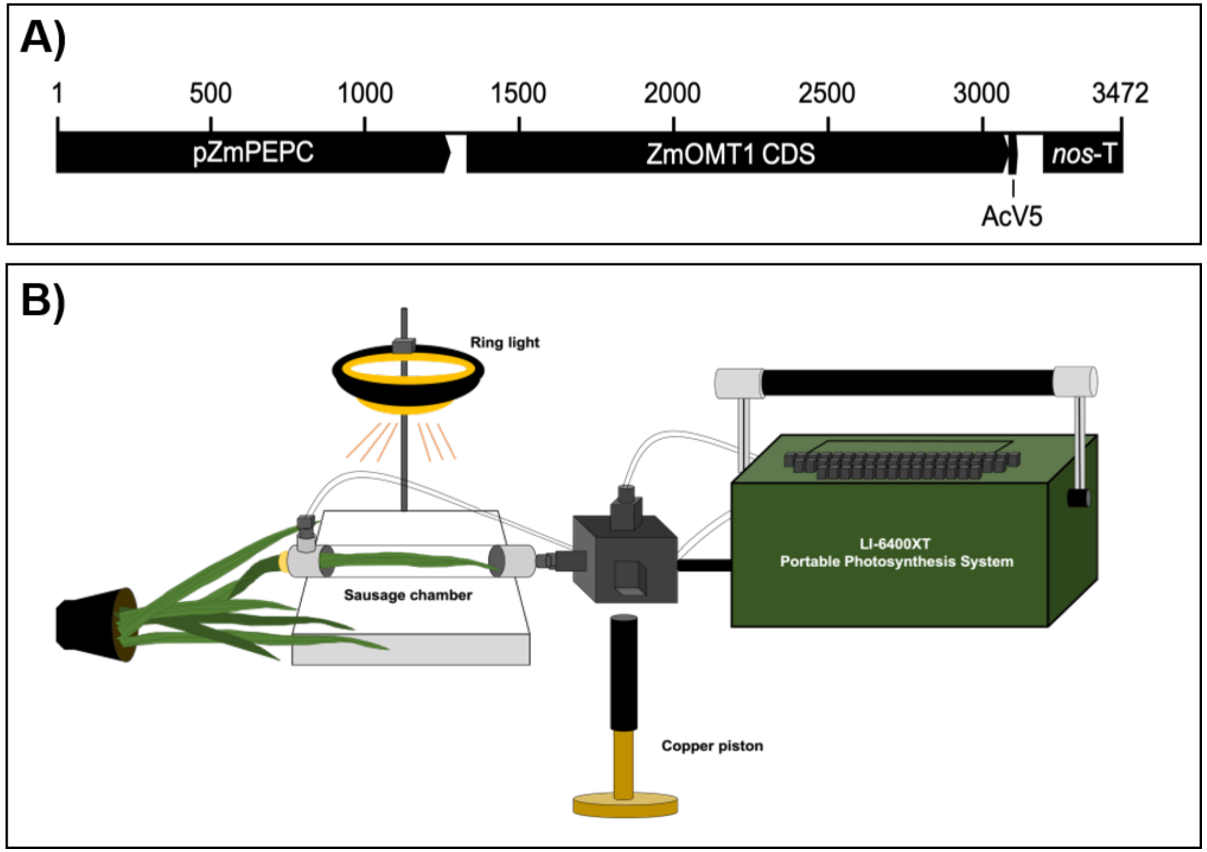

Fig. S1: Schematic of the pSC110:*ZmOMT1*:AcV5 construct **(A)**. Schematic view of the customized gas exchange assembly and its coupling to the LI-COR 6400-Protable Photosynthesis System **(B)**.

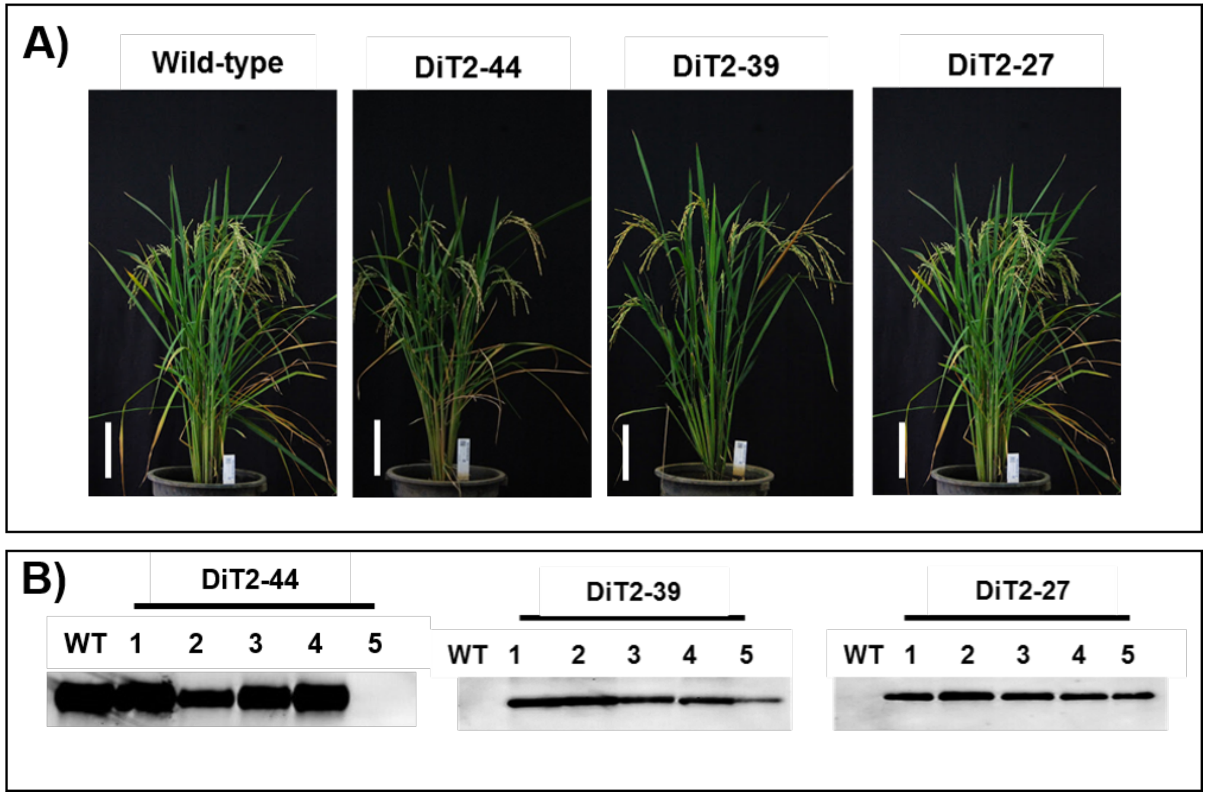

Fig. S2: Representative pictures of wild-type, DiT2-44, DiT2-39 and DiT2-27 lines grown under ambient conditions; 80 days post germination (DPG). The MW of DiT2-AcV5 is 40 kDa.Scale bar: 15 cm **(A)**. Western-blot analysis of *ZmDiT2*-AcV5 protein expression in rice DiT2-44, DiT2-39 and DiT2-27 leaves at mid-tilling stage. WT, wild-type plants **(B)**.

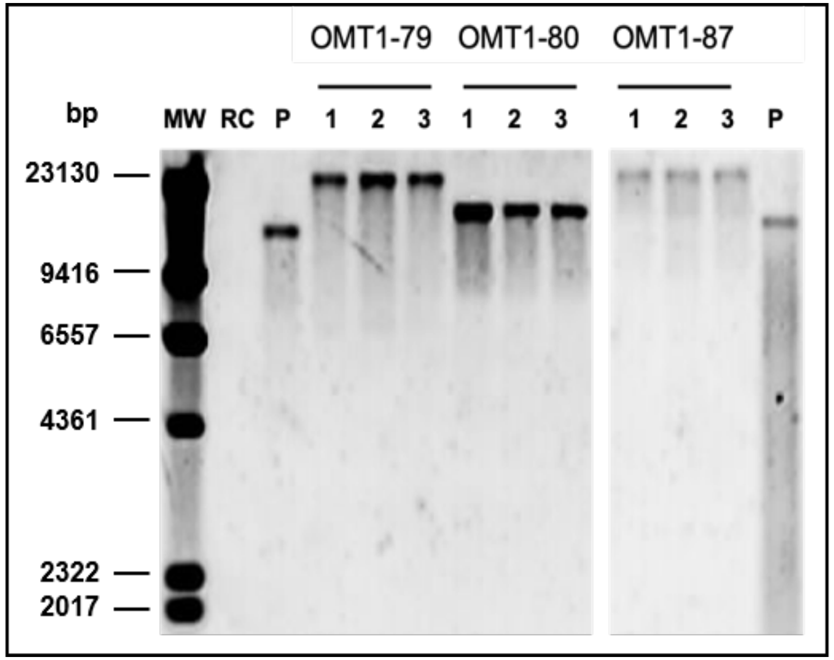

Fig. S3: DNA blots show that the OMT1 lines (OMT1-79, OMT1-80 and OMT1-87) carry a single copy of the *ZmOMT1* CDS and are homozygous at the T_3 generation. Untransformed rice control plants (RC) are negative controls for the transgene. The vector plasmid (P) is a positive control. A DIG-labeled DNA molecular marker indicates molecular weight (MW).

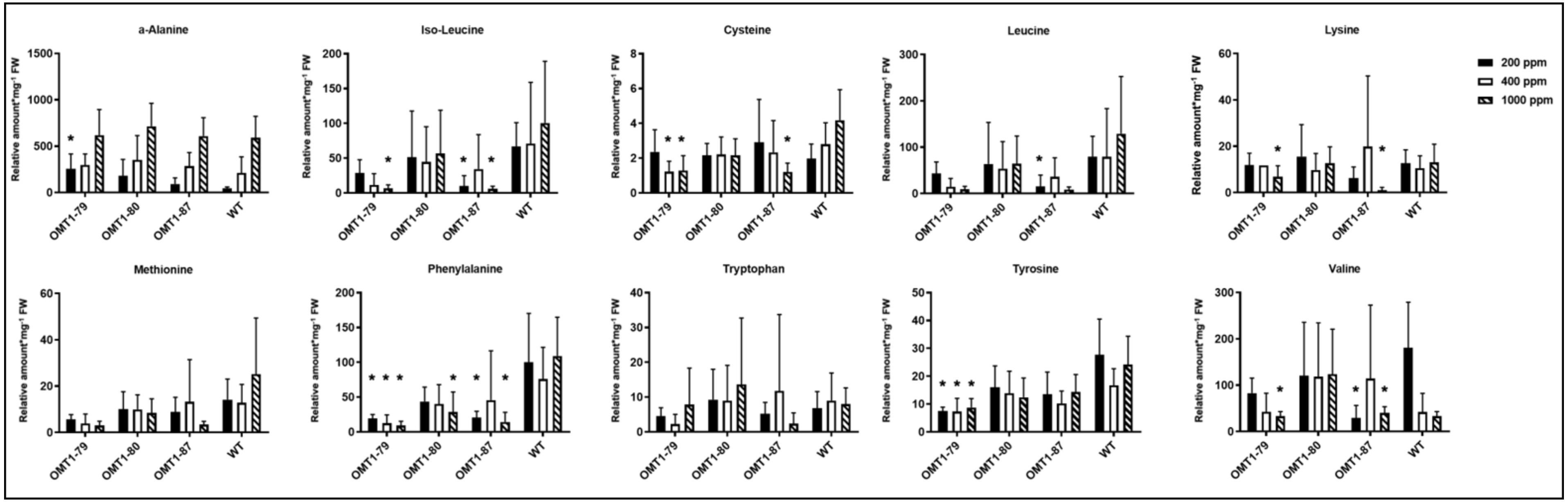

Fig. S4: Relative amount of some individual amino acids in OMT1 lines (OMT1-79, OMT1-80 and OMT1-87) and wild-type (WT) plants under different CO_2 concentrations (200, 400 and 1000 ppm). Value represent the mean ± SEM, n=5, significantly differences are indicated * $P \leq 0.05$, Student's t-test

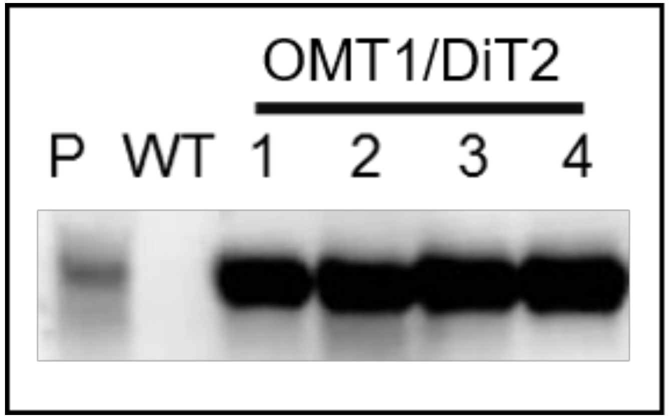

Fig. S5: Western blot analysis of *Zm*OMT1 and *Zm*DiT2 protein expressions in rice crosses lines of OMT1/ DiT2 (1, 2, 3 and 4). P; OMT1-AcV5 plant (positive control). WT; wild-type plant (negative control).

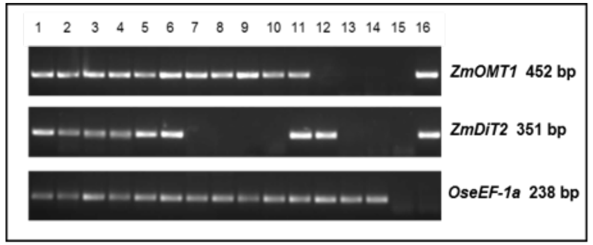

Well No.	Sample
1	OMT1-79/DiT2-44-1
2	OMT1-79/DiT2-44-2
3	OMT1-45/DiT2-27-1
4	OMT1-45/DiT2-27-2
5	OMT1-80/DiT2-39-1
6	OMT1-80/DiT2-39-2
7	OMT1-79-1
8	OMT1-79-2
9	OMT1-45-1
10	OMT1-80-1
11	OMT1-79/DiT2-44-3
12	DiT2-44-1
13	WT
14	WT
15	Water
16	Plasmids of ZmOMT1 and ZmDiT2

Figure. S6: RT-PCR of *ZmOMT1* and *ZmDiT2* mRNA expressions in OMT1/DiT2 double transgenic lines together with wild-type (WT). RT-PCR of rice housekeeping gene *OseEF-1a* was used as a positive and quality control. The PCR condition were: 95°C for 3 min; 40 cycles of 95°C for 20 sec, 55°C for 30 sec and 72°C for 45 sec; and 72°C for 3 min. RNA was extracted from leaf materials using TRIzol reagent and treated with DNase and the PCR products were electrophoresed on 2% agarose gel.

Supplementary Tables

Table S1. Primers used in this study

Target Gene	Forward Primer	Reverse Primer
ZmPEPC promoter (GRMZM2G083841; base pairs –1212 to +1)	CACCATGGTCGACGCGTCCTCCAC	TCAAGACCAGCCGCTCGCATCTTTCCAAGACCACAGCCCGATTATCTTC
ZmOMT1 for PCR screening	CGTGGGATACCCTTACATGG	CCCGATTATCTTCCACCAGA
OsOMT1 for qPCR	ATGGAATTGGGTCTGCTCCTG	AATCCATACCCCCACCACTG
ZmOMT1 for qRT-PCR	GTGGGGCTATGGGTTTGTCA	TATCTTCCACCAGAAGCCGC
ZmOMT1 for RT-PCR	CGTGGGATACCCTTACATGG	CCCGATTATCTTCCACCAGA
ZmDiT2 for RT-PCR	GTTGGAATGGCAGGACAACT	ACCCAGCCTGAAAACATCTG
OseEF-1a for RT-PCR control	CAACATTGTGGTCATTGGCC	GCAGTAGTACTTGGTGGTCT
ZmPEPC-promoter for digoxigenin labelled probe	TCCCGAGTTCCTAACCACAG	GTGGCTGAGGCTTCTTTTTG
Cloning of *ZmDiT2* for overexpression in rice	CACCATGGAGCTCCACCTCGCCAC	TCAAGACCAGCCGCTCGCATCTTTCCAAGAGTACAGACCCAAAAATTTCCACCAGATG

Table S2: Vcmax and Jmax based on ACi Data at 21% or 2% O_2 using the PsFit Model.

	**Vcmax 25°C** (μmol m^{-2} s^{-1})	**Jmax 25°C** (μmol m^{-2} s^{-1})	**Vcmax 25°C** (μmol m^{-2} s^{-1})	**Jmax 25°C** (μmol m^{-2} s^{-1})
	**21% O_2**		**2% O_2**	
**Wild-type**	93.77 ± 7.82^{a}	176.18 ± 18.19^{a}	121.7 ± 10.91^{a}	154.77 ± 11.58^{ab}
**OMT1-79**	65.06 ± 5.75^{bc}	140.78 ± 6.59^{ab}	72.41 ± 11.28^{b}	114.52 ± 8.86^{c}
**OMT1-80**	86.47 ± 12.59^{ab}	167.72 ± 20.71^{a}	124.68 ± 25.39^{a}	171.03 ± 20.73^{a}
**OMT1-87**	58.59 ± 14.15^{c}	122.48 ± 20.16^{b}	100.04 ± 18.48^{ab}	126.45 ± 21.21^{bc}

Vcmax, maximum rate of Rubisco carboxylation allowed by Rubisco; Jmax, maximum rate of electron transport. Value represent the mean ± SEM, n=5. Different letters within groups indicate that values are statistically different $p \leq 0.05$, Tukey's multiple comparison test. ns indicates non-significant, $p > 0.05$.
